# Literary Fools.—Bluet D'Arbères
*"Études Bio-Bibliographiques sur les Fous Litteraires." Par Octave Delepierre.—(*Privately printed*.)


**Published:** 1859-01-01

**Authors:** 


### THE JOURNAL

OF

### PSYCHOLOGICAL MEDICINE

AND

### MENTAL PATHOLOGY.

JANUARY 1, 1859.

### Art. I.—LITERARY FOOLS.—BLUET D'ARBÈRES.*

Fool is a technical as well as a conventional word. In its conventional signification it is familiarly known and widely and freely used; in its technical signification it expresses a form of mental disorder, which has been aptly considered by Feuchtersleben as in some measure the prototype of other forms; but the affinity which exists between the conventional and technical meanings of the word is invariably more or less conspicuous even in the freest usage to which it is put in the affairs of common life.

The most marked characteristic of the fool, in the technical sense of the term, is a peculiar abnormal mobility of thought and emotion, which parallels in the mind the erratic and ludicrous movements that are observed in the muscles in choreatic affections—as, for example, in St. Vitus's Dance. In whatever manner the intellectual faculties and the emotions are called into play there is found a preponderance of their automatic manifestations. This is co-existent with, and it is indeed significant of a weakened volitional power, and a deficiency of co-ordination in the mental faculties. The thoughts, imperfectly controlled by the will, hasten along as in the consistent-inconsistency of dreaming, and they are reflected in the voluble tongue and restless actions; while the emotions rapidly succeed each other, joy alternating with grief, anger with fear, upon the most trifling incitements.

In the slighter, connate forms of the disease the abnormal mobility of thought is shown in the eccentric and fantastic associations of the ideas which throng the mind. Every thought and sensation excites an anomalous sequence of ideas, which commonly present actions and events in a ludicrous light. This species

* "Études Bio-Bibliographiques sur les Fous Litteraires." Par Octave Delepierre.—(*Privately printed.*)

of folly is quite consistent with considerable powers of perception and observation; but it is never dissociated with those general indications of weakened intellectual power which are summed up in the expressive word *foolishness.* It was for this rarer form of folly that the Court fools of the middle-ages were chiefly distinguished; and the fantastic nimbleness of fancy which characterizes it have been fixed indelibly in language, by Shakspeare, in the characters of the Fool in "King Lear," the Clown in "Twelfth Night, or What You Will," and Touchstone, in "All's Well that Ends Well."

In the more highly-developed forms of folly disconnected ideas course in rapid succession through the mind, crowding one upon the other in a confused and tumultuous manner, and the emotions change with, and as rapidly as, the conceptions. Hence, in fools of this class, a general craziness of thought is observed, an overwhelming, senseless loquacity, and a motiveless bustling activity. They are forgetful, volatile, inconsiderate, and incapable of reasoning; and their passions are easily excited, readily calmed, and rarely terminate in violence.

There are many modifications of folly, and it is connected by insensible gradations with idiocy on the one hand and mania on the other. Hallucinations are present in nearly every form of the disease, and delusions are probably of more frequent occurrence than is commonly supposed.

The technical, rather than the conventional meaning of the term, is most applicable to the word "fool" as it is used in the Essay which forms the subject of this article. Bluet d'Arbères, to an account of whose life and writings the Essay is devoted, was a fool of some little note in his day. The most marked phase of his folly was exhibited in certain strange literary productions, the chief value of which, at the present time, arises from the curious psychological study they afford, and from the little flecks of light they cast upon the social economy of France and Savoy during the five last lustres of the sixteenth, and first lustre of the seventeenth centuries. The records of Bluet d'Arbères' acts and doings have been disentombed and reduced into a reasonable compass by M. Delepierre, with a delightful pseudo-serious unction; and bibliographer, historian, and psychologist, will dwell with rapture upon the daintily-set gem of literature which he has presented to them.

This, with a few additions, is M. Delepierre's story:—In the year of Grace, 1566, there was born, of poor parents, in the village of Arbères, near Divonre, in the territory of Gex, Switzerland, one Bernard de Bluet. As a lad he tended flocks, and very early in life he believed that Providence destined him to play an important part in the world. He tells us himself, in his curious autobiography, that the village of his birth was situated in the lowlands, and that towards the sun-setting there were grand ranges of mountains where rocks and sweet-smelling herbs alone could be seen; and that towards the sun-rising there were but swamps. He tells us also that he remembered all that he had done and said even from the cradle. When an infant he was held in the arms by one of the great men of the village, and as soon as he could walk he began to climb upon the great coffers of the peasants, and sing in a loud voice to the Lord. It was a custom of the peasants who had sown millet to place images of Christ in the fields, in order to scare the birds. These images Bluet was in the habit of stealing from the great desire that he had to know God. When he began to tend sheep a wolf attacked them, but he cried to God and the ravenous beast fled. Even a companion who had played him a foul trick, while he slept, was constrained to confess the fault, and forthwith the offender died. These events happened in the first lustre of Bluet's life.

So great was the influence of his childish prayers, that, so long as he had charge of sheep, they were safe from the wolf, but if his father took charge of them they were at once attacked. Moreover, he recounts, that being cold in the month of March, he prayed that the clouds which hid the face of the sun might be removed, and God in answer to his prayer dispersed them. His mind was filled with the desire to become a preacher from the respect in which learned men were held, and from a wish (in a great measure prompted by vanity) to teach his companions, and he prayed constantly to God that he might have knowledge and science. The gifts and graces of David and Moses made a great impression upon his childish mind; but, alas! there steals out of his account, at every turn, the painful fact that his solitary musings and vague desires were determined in no small degree from his being scouted and laughed at by children of his own age for his simplicity and foolishness.

Before the termination of his second lustre his father wished him to take charge of a herd of cows. To this Bluet objected, as he conceived that the cow was not so worthy a beast as a sheep, that being the most worthy of all animals except the dove. Compelled, however, by necessity he began to tend kine, and he naïvely tells us that he found it a less devotional duty than that of tending sheep, as the cows were never attacked by the wolf, and hence there was not so great an inducement to devotion. His mind, therefore, being less pre-occupied by holy thoughts, he was persecuted by his feelings to love unchastely the peasant girls. He was an enemy to all other vices, but he found that this was the most pleasant vice.

Before his twelfth year he prophesied that the country about

Geneva would become the seat of war; and he told his companions that, when he became a great man, they would see him in the suite of princes, and afterwards of kings, and if it pleased God he should wear habits like unto theirs, satin and velvet, tricked out with gold.

In the midst of vague childish dreams he longed with impatience to distinguish himself by some warlike exploit. He made cuirasses of the bark of trees, morions of pumpkins, sabres, arquebuses, and pistols of wood, &c., with which he proposed to arm his companions, and to conduct them to some prince who might desire to have their services; and with the profits obtained by the sale of baskets at Geneva he bought taffeta, from which he made ensigns of war. When these preparations were completed, he communicated his project to those of his comrades whom he placed most confidence in. He afterwards distributed the arms to them, and conferred upon each of them a title of nobility, and declared himself to be their chief, without asking their consent. The boys and girls of the village fanned Bluet's notions by adopting them and paying court to him; but he tells us that when he first made known his great preparations and high ambition, his parents took their stand near him, and weeping exclaimed, "that he did them shame, and they had rather have nourished a pig than him." But he told them that they dishonoured him, and that he would be an honour to them, and heap them with favours, whilst he would be disgraced by them.

Believing that it was beneath him to gain a livelihood by the work of his hands, he fled from home (when he was about twelve years of age, according to his own story). One of the principal inhabitants of Rumilly received him, from charity, into his house, and as he stated that it was his intention to marry, this was made a plea to decide him in selecting an occupation which would give him an opportunity of bringing up a family when he might have one.

He then undertook the trade of wheelwright, and he was employed for some time in mounting cannon, at the Fort of the Annonciade, in Savoy. As soon, however, as he had obtained a little money, he dressed himself in carnation-coloured garments, placed a feather in his cap, and with a sword at his side and poniard in his girdle, he hastened to his native village to show himself, thus accoutred, to his poor comrades. The compliments they lavished upon him, on account of his brilliant equipment, still further unsettled his brain; he assured them of his protection, and believing that he had become an important personage, he dubbed himself *Superintendent of Artillery-mounters of the Castle of the Annonciade.*

While at Rumilly he was occasionally admitted to the tables of the gentry, and he enacted there the character of Fool, although he attributes his admission to an acknowledgment of his genius and talents.

He quitted Rumilly when sixteen years of age, and offered his services to the governor of the citadel of Montmélian, who consented to give him employment. His vanity exposed him in this city to many misadventures, which he recounts very naïvely, but always having care to give them a creditable aspect in so far as he was concerned. Angered, however, by the tricks which his comrades made him the victim of, he left Montmélian, and after having wandered some time in the neighbourhood of Chambery, leading a very austere life, in order to reduce his temperament, he set out again for Arbères, and announced himself there as a prophet sent from God to convert the Philistines, as he termed the Protestants. Bluet had been born and baptised a Protestant, but whilst living at Rumilly he had embraced the Romanist doctrines The announcement of his prophetic mission at Arbères not having produced the effect he intended, he shook the dust from his feet, and, in 1597, sought the Duke of Savoy, at Chambery. This prince (who is named King David by Bluet in his writings), being amused at his extravagances, clothed him in his livery, and assigned him a maintenance. In the suite of this Prince, Bluet travelled through Piedmont, and saw Alessandria, Asti, and Turin, where he passed several years, serving as a butt for the pleasantries of the courtiers. They had persuaded him without difficulty that all the demoiselles of Turin contended for the happiness of pleasing him; but he had given the preference to the mistress of the Duke of Savoy, and he carried publicly her colours. One day, when he was upon his knees before this lady, the duke caused him to be seized by four lackeys, and tossed in a blanket, like the unfortunate squire of Don Quixote. This discourteous treatment displeased him, and he demanded his *congé*, which he obtained without difficulty. He went into France to see the great Emperor Theodosius (as he termed Henri Quatre), who, however, did nothing for him.

When thirty-four years of age, Bluet began to publish his lucubrations, in the form of small pamphlets or fly-sheets, of which upwards of one hundred are known to bibliographers. These sheets contain a curious collection of fantastic and incoherent visions and dreams, devotional exercises, and many particulars of Bluet's life. Religious delusions form the most notable characteristics of the different writings, and much lasciviousness of thought is found in them; but a certain degree of shrewdnes crops out here and there.

In the title to his collected works Bluet writes:—

"*The* INTITULATION *and collection of all the works of Bernard de Bluet D'Arbères, Count by Permission, Chevalier of the XIII. Con-*

*federated Swiss Cantons: the said Count by Permission gives you to understand that he knows not, neither has he ever known, how to read or to write, except by the inspiration of God, and the guidance of Angels, and for the goodness and mercy of God. And the whole shall be dedicated to the high and puissant Henry of Bourbon, King of France and of Navarre, great Emperor Theodosius, chiefest son of the Church, Monarch of the Gauls, the first of the world, by the grace, goodness, and mercy of God.*

*This is to make declaration of the books which have been printed in his name, which have had their fulfilment, reserving three of all my works, until it pleaseth God to call me. And there shall be given, concerning all my works bound in one, declarations to all the governors and great lords of the earth who are my friends, and it* (sic) *shall be dated the day and the time that they shall have received and printed them, and shall be taken for a testimony to declare the truth of the visions which have not yet had their fulfilment, and to declare the truth of those which shall have fulfilment if it please God. May,* 1600, *in* 12°."

Equally curious and significant of mental disorder is the title which heads his Book of Orisons. It runs thus :—

"ORISONS, *which have been given to Bernard de Bluet d'Arbères, Count by Permission, by the inflammation and inspiration of the Holy Ghost and of the Angels: they were not given to him when he frequented the world, but when he frequented the catacombs* (*testes des mortes*) *at Meing, near Chambery, which is the most ancient church of Savoy, and the solitary places, and not for his good deeds, but according to the grace and goodness and mercy of the holy court celestial.*"

The amusing character of Bluet's egotism is well shown in one of his visions :—

"It appeared to me," he writes, "that I was transported to the house of a great lady, one of my friends. I was dressed in an antique habit, and carried a *palle de feu* in my hand. There was a table covered with vessels of silver-gilt. . . Three Capuchins who had resplendent (*reflambante*) faces said to the company that they had come to see me. I spoke to them, the tears distilling from their eyes, and they said to me, 'You have the very highest obligation from the Great God on high; there never was, and there never will be, a Pope able to do this which you have done. Your books will reign even to the end of the world; you will be regarded as a wonder in the future, which you are not now; show us your works.' I showed them. When they had (seen) them, they commenced to sing in a loud voice, 'Glory be given to the great God Eternal, and blessings be upon your actions and your works.' I said to them, 'This is nothing in comparison to that which I shall do in the future, if it please God. I am about to remove all the difficulties of all the divisions, including Turkey, &c.'"

In Paris Bluet led the life of a literary vagabond, barely subsisting and meanly clad, standing at the doors of the great to present his books in hope of receiving a handsome gift in return, and spending such money as he did obtain mainly in printing his sheets. At length he died; and in the manner of his death the Fool became ennobled. The plague broke out in Paris in 1603, and ravaged the city for several years, at the acme of the outbreak the deaths numbering two thousand every day. About the year 1606, Bluet conceived that it was his duty to intercede with God by prayer and fasting, and to offer up his life as an expiatory sacrifice for the plague-stricken city. He retired to the cemetery of St. Etienne, and there, amidst the tombs, rapt in devotion, he fasted; and on the fifteenth day he died, happy in the thought that his death would stay the pestilence.

Such is an outline of the life and character of Bluet d'Arbères. The peculiarities which distinguished his folly differed little from childhood to the grave. The childish personal vanity which he exhibited in the first two lustres of his life clung to him until death, deepening merely as he advanced in life into a more absorbing egotism. His vanity prompted the most fantastic phases of his love-dreams and amorous delusions; but these were mainly induced by an ungovernable lasciviousness of thought which manifested itself first in childhood. That eccentric fashion in which his vanity showed itself when a child, by the mode in which he attached names of nobility to himself and comrades, was exhibited also at every period of life in the divers titles he from time to time assumed; and somewhat akin to this grotesque fancy was the passion he displayed to attach symbolical names or titles to those persons of dignity with whom he was thrown into contact. The childish longing for religious distinction which he indulged in while tending his flocks formed the substratum of those notions of inspiration which dominated the major part of his life and acts, and which culminated in the delusion which ended his days.

Bluet d'Arbères was not the solitary fool of his time. A contemporary writer ranks him among a number of madmen who, in the epoch preceding the civil wars in France, wandered from city to city. These men, bearded and having dishevelled hair, filthy and half-naked, recounted to all they met in the market-places and public resorts the "fantasies of their black frenzy," from the morning until the setting of the sun.

The religious notions which were dominant in the delusions of Bluet d'Arbères were but the reflex of the sole absorbing general feeling of the days in which he lived. The year in which he first saw the light is seared with the scheme which was concerted between Catherine of Medicis and Philip of Spain for the total extermination of the Protestants by fire and sword. Bluet d'Arbères lived through the wars which tore up France and the

Low Countries with the horrible mitraille of religious discord; and the enthusiastic, unrestrained nature of the religious opinions held in those days are shown in his delusions and writings. He was sufficiently shrewd to see the folly of the struggles between Huguenot and Catholic, and in his 21st Book he writes:—

"There are the preachers of both religions: the most part of their preaching is to incite the professors of the one religion to cut the throats of those of the other. The Protestant preacher preaches that the poor papists make a God of paste and a silver goblet: they are idolaters. The Catholic preachers say that the Calvinists are dogs who eat flesh at all times. The Count by Permission gives you to understand on the part of God that it is not well to retail all these words. . . . that of thirty thousand who go to the church there is not one who does his duty."

Bluet d'Arbères was from birth a fool, and as such his contemporaries held him; but his works have been the cause of foolishness, in the conventional sense of the term, in others. Writings exist of men who have thought that they have discovered in this poor fool's works marks of true inspiration, or of the occult analysis which would lead to the discovery of the philosopher's stone. The faith which Bluet d'Arbères reposed in his inspiration, and the prophetic character of his visions and dreams, was doubtless indulged also by several individuals in his own time, as it will probably be by some in ours. The divinely-prophetic power of the madman is no new belief, and Ennemoser, in his work on "Magic," not long ago published in an English guise, by Mr. Howitt, quotes two instances of the prophetic power of fools. One example will suffice:—

"Claus, the fool, at Weimar suddenly entered the privy council, and exclaimed, 'There you are all, consulting about very weighty things, no doubt; but no one considers how the fire in Coburg is to be extinguished.'' It was afterwards discovered that a fire had been raging at the very time in Coburg."*

We fear that M. Delepierre will have his eyes upon M. Ennemoser and his writings.

Literary fools are of no specific age and date. It would perhaps not be difficult, even in these days, to lay hands on works from which the freshness is scarcely worn off, but which have a marked similitude to Bluet d'Arbères' writings. M. Delepierre quotes many instances of literary fools in past centuries, and he wickedly hints that Kant and Hegel (if the anecdotes told of them are to be believed) are not so widely separated from the class of which Bluet d'Arbères will become in future a representative, that they may escape altogether outside its boun-

* "The History of Magic." By Joseph Ennemoser. Translated from the German by William Howitt. Bohn. Vol. i. p. 80.

daries. M. Delepierre, however, promises in due time to furnish us with essays on the whole of the distinguished literary fools and eccentrics of history, and we shall wait with impatience for the second part of that series of which Bluet d'Arbères forms so fascinating an introduction.

---

### Art. II.—ON PUERPERAL INSANITY.

